# The efficacy of NP11-4-derived immunotoxin scFv-artesunate in reducing hepatic fibrosis induced *by Schistosoma japonicum* in mice^☆^

**DOI:** 10.1016/S1674-8301(11)60019-5

**Published:** 2011-03

**Authors:** Hong Li, Chunyan Gu, Yongya Ren, Yang Dai, Xiaojuan Zhu, Jing Xu, Yuhua Li, Zhenning Qiu, Jin Zhu, Yinchang Zhu, Xiaohong Guan, Zhenqing Feng

**Affiliations:** a Key Labortary of Antibody Technique of Ministry of Health, Nanjing Medical University, Nanjing, Jiangsu 210029, China; b Pathology Department, Nanjing Medical University, Nanjing, Jiangsu 210029, China; c Jiangsu Institute of Parasitic Diseases, Wuxi, Jiangsu 214000, China; d Huadong Medical Institute of Biotechniques, Nanjing, Jiangsu 210002, China

**Keywords:** *Schistosoma japonicum*, scFv, immunotoxin, hepatic fibrosis

## Abstract

Schistosomiasis is one of the most prevalent parasitic diseases in China, and hepatic fibrosis caused by schistosome infection is the principal cause of death. The aim of this study was to evaluate the efficacy of NP11-4-derived immunotoxin scFv-artesunate on *Schistosoma japonicum*-induced hepatic fibrosis. A single-chain variable fragment (scFv) was generated from the murine anti-*Schistosoma japonicum (S. japanicum)* monoclonal antibody NP11-4. The scFv was expressed as a soluble protein and purified by Ni-affinity chromatography. After conjugation with artesunate, the binding ability with soluble egg antigens (SEA) was determined by an enzyme-linked immunosorbent assay (ELISA). The biological activity of purified scFv, scFv-artesunate (immunotoxin), and artesunate was detected *in vivo*. Image-Pro Plus software was used to analyze the size of egg granuloma and the extent of liver fibrosis. The recombinant scFv expession vector was constructed and expressed successfully. After purification by a His-trap Ni-affinity column, the scFv yield was approximately 0.8 mg/L of culture medium. ELISA results showed that chemical conjugation did not affect the binding activity of the immunotoxin. Our animal experiments indicated that the immunotoxin could significantly reduce the size of egg granuloma in the liver and inhibit hepatic fibrosis. The immunotoxin could be used as a promising candidate in the targeted therapy of *S. japonicum*-induced hepatic fibrosis.

## INTRODUCTION

Schistosomiasis, which is caused by several species of the trematode *Schistosoma*, is an endemic disease in 74 developing countries. In individuals infected with *S. japonicum*, chronic egg-induced inflammation in the periportal tracts of the liver can lead to fibrosis, portal hypertension, bleeding, and eventually death[1]–[4]. In China, schistosomiasis japonica is endemic in the 12 provinces along the southern part of the Yangtze River[5]. The estimated number of cases of chronic schistosomiasis japonica in 2008 is 412,927, which represents a great reduction from the 690,000 reported cases for 2000[6]–[8]. Recently, the disease has reemerged in many places where it was previously under control. Unlike other infectious diseases, re-infection with schistosomes can occur even after successful chemotherapy[9].

Over the past 30 years, praziquantel has been widely used for the treatment of patients infected with *S. japanicum* in China. With its high efficacy and low cost, it has become the backbone of the national schistosomiasis control program in China[10]. However, many studies have shown the existence of strains were resistant to anti-schistosome agents, including oxamniquine and praziquantel[11]–[14]. Deaths caused by acute schistosome infection still occur, while no effective treatment has yet become available for chronic schistosomiasis-induced liver fibrosis. Therefore, a new treatment for schistosomiasis is required.

Immunotoxins, which couple the specificity of monoclonal antibodies (MAbs) with a highly lethal cellular toxin, have been used to selectively eliminate cell subpopulations *in vivo*[15],[16]. The murine anti-*S.japonicum* monoclonal antibody NP11-4 was prepared in our previous work[17]. It was an antibody binding to the membranes of cercariae, adult worms, and eggshells. As a murine monoclonal antibody, human anti-mouse antibody (HAMA) reaction limits its clinical applications. In this study, we describe the generation of a scFv by isolating the genes encoding heavy (V_H_) and light (V_L_) chain variable regions from the NP11-4 hybridoma. The immunotoxin was constructed by chemically conjugating scFv with artesunate, and its binding activity was evaluated by enzyme-linked immunosorbent assay ELISA *in vitro* . The therapeutic efficacy of immunotoxin for schistosomiasis-induced liver fibrosis was determined *in vivo*.

## MATERIALS AND METHODS

### Cloning of scFv genes

Hybridoma cell lines NP11-4, which produced the monoclonal antibody against *S. japonicum*, was previously established in our laboratory. The sequences of *V_H_* and *V_L_* gene were obtained in the previous work[18],[19]. Total RNA was isolated from about 1×10^7^ fresh NP11-4 hybridoma cells, using the RNA Now kit (Promega, USA). First-strand cDNA was synthesized from total RNA using MMLV reverse transcriptase and oligo (dT) primer (Takara, Japan) according to the manufacturer's protocol. The V_H_ regions were amplified with the primers V_H_F and V_H_R. The primers V_L_F and V_L_R were used for amplifing V_L_ regions. The sequences of the different oligodeoxynucleotide primers are listed in ***Table 1***. The V_H_ gene of anti-*S. japonicum* was fused to the 5′ end of the V_L_ gene *via* an 18-amino acid linker sequence (GGSSRSSSSGGGGSGGGG) using the overlapping PCR method to generate the anti-*S. japonicum* scFv gene. The amplified scFv gene was cloned into the pMD18-T vector (Takara, Japan) and sequenced by Bioasia Co., Ltd (Shanghai, China).

**Table 1:** Primer sequences for construction and sequencing of scFv antibody

Primers	Sequence (5′ → 3′)
V_H_F	CGCCATGGGATCCCTCGAGGTCCAGCTGCAGCAG
V_H_R	GGAAGATCTAGAGGAACCACCTGCAGAGACAGTGACCAGAGTCC
V_L_F	**GGTGGTTCCTCTAGATCTTCCTCCTCTGGTGGCGGTGGCTCGGGCGGTGGT**
	**GGG**GAGCTCGATATTTTGATGACCC
V_L_R	GGAATTCCAGGACCGCCTCCTGGTTTGATTTCCAGTTTGGTCCC

Restriction sites *Nco* I and *EcoR* I are underlined. Boldface indicates the DNA sequences used to integrate an 18-residue linker.

### Construction and expression of recombinant plasmid

The amplified scFv gene was digested by *Nco*I and *Eco*RI restriction enzymes, and then inserted into the pBAD/g III A vector. *E. coli* Top10F′, transformed with the recombinant plasmid pBAD/gIII A-scFv was grown in a shaking culture (220 *g*, 37°C) in a 2×YT medium supplemented with 100 µg/mL ampicillin to an OD_600_ of 0.8-1.0. Protein expression was induced by the addition of 0.02% L-arabinose (Sigma, USA) and 4% sucrose for 20 h at 23°C at 220 *g*. The cells were centrifuged at 10,000 *g* for 10 min at 4°C; the pellet was resuspended in phosphate buffered saline (PBS) to 1/10 of the volume of the original culture and left for 30 min on ice with sonication. After centrifugation at 12,000 *g* for 30 min at 4°C, the supernatant containing soluble periplasmic proteins was collected and analyzed by SDS-PAGE and Western blotting.

### Purification and concentration of scFv

ScFv was purified by a His-trap Ni-affinity column and AKTApurifier^™^ system according to the manufacturer's protocol. The periplasmic fraction was filtered through a 0.22 µm filter, and loaded on a His-trap Ni-affinity column. Then the Ni-affinity column was equilibrated with 10 column volumes of binding buffer containing 50 mmol/L PBS, 0.5 mol/L NaCl, and 20 mmol/L imidazole. The protein of interest was eluted with the same buffer-added imidazole to 50, 100, 200, 300 and 400 mmol/L, respectively. SDS-PAGE showed that scFv was mainly eluted by a 100 mmol/L imidazole. After that, the scFv was concentrated to 1 mg/mL utilizing an amicon ultra centrifugal filter device with a 10 kD cut-off (Millipore).

### Immunotoxin fabrication by chemically conjugating scFv with artesunate

Ten mg artesunate was dissolved into 5 mL PBS, and 10 mg couplant A was dissolved into 1 mL dimethylformamide (DMF), and then incubated in an artesunate-couplant A mixture at 4°C for 6 h. Then 4.4 mg couplant B was dissolved in 1 mL DMF, and scFv was dissolved in 2 mL PBS. Next, The scFv-couplant B mixture was incubated at room temperature for 1 h. Subsequently, the artesunate-couplant A and scFv-couplant B were mixed 1:1 and incubated at room temperature for 1 h. The scFv-artesunate conjugation (immunotoxin) was obtained by changing the buffer system into PBS. The maximum absorption peak of immunotoxin was detected to determine whether it was conjugated.

### Assay of binding activity by indirect ELISA

For detection of the binding ability of scFv and the immunotoxin in combination with solube egg antigens (SEA), ELISA was performed by coating the 96-well plate with SEA at 10 µg/mL diluted in a sodium carbonate buffer (pH 9.6) and incubating the plate overnight at 4°C. The wells were blocked with 1% bovine serum albumin (BSA) in PBS. The primary antibodies were the purified scFv and immunotoxin diluted to 40 µg/mL with 5% non-fat milk in PBS. The secondary antibody was an anti-His antibody conjugated with peroxidase. Each step was followed by five washes with PBS plus 0.05% Tween20 (PBST). All of the immunocomplexes were revealed with 3, 3′, 5, 5′-tetramethylbenzidine (TMB) and read at *A*_450_.

### Animal experiments

The therapeutic effect of the immunotoxin on the *S. japonicum* infection was assayed by animal experiments. Eight-week-old BALB/c mice (18-22 g) were purchased from Shanghai SLAC Laboratory Animal Co., Ltd. All mice were maintained in pathogen-free conditions and treated with standard laboratory food and water. Snails were bred and maintained in the laboratories of the Institute for Parasitic Diseases of Jiangsu Province.

Sixty male BALB/c mice were infected with 25±2 cercariae of *S. japonicum* through abdominal skin and divided into 4 groups (15 mice/group): the immunotoxin group (immunotoxin 5 mg/kg), the scFv group (purified scFv 5 mg/kg), the artesunate group (artesunate 5 mg/kg) and the negative group (PBS). Immunotoxin, purified scFv, artesunate and PBS, respectively, were injected into the quadriceps femoris muscle on d 1, 14, and 28 after infection. Ten weeks after infection, the surviving mice were sacrificed and their livers were isolated for histological studies.

### Histopathologic analysis

Liver samples were fixed in 10% buffered formalin and processed for routine histopathological analysis. Four-µm sections were stained with H&E and Masson. To determine more accurately the true shape and dimension of granulomas, only those with a visible central egg were counted. A minimum of 10 granulomas were scored per liver section. The size of egg granuloma and the integrated optical density (IOD) of hepatic fibrosis were determined by computer-assisted morphometric analysis using Image-Pro Plus software (Media Cybernetics, USA).

### Statistical analysis

All data in the study were evaluated with the SPSS 13.0 software. One-Way ANOVA and Student-Newman-Keuls (SNK) tests were used to determine the statistical significance. A *P* < 0.05 was considered statistically significant.

## RESULTS

### Amplification of the V_H_ and V_L_ regions of NP11-4 monoclonal antibody and construction of the scFv gene

**Fig. 1:**
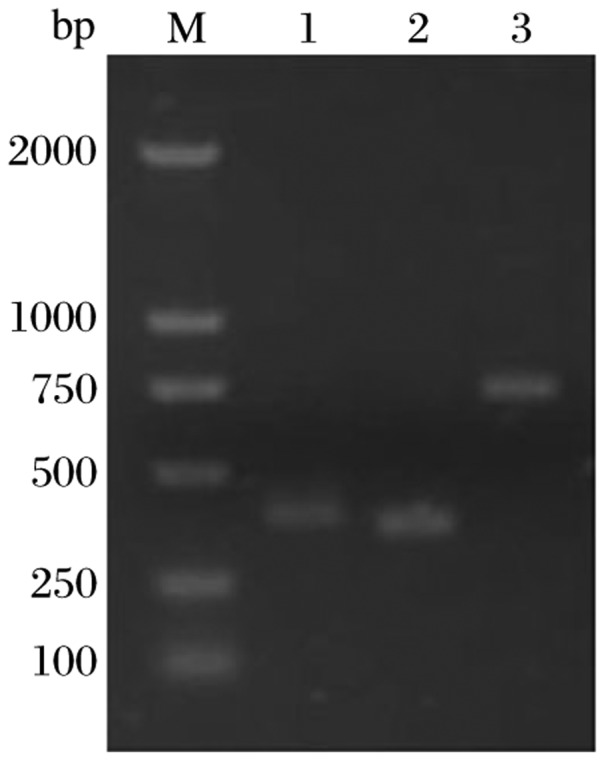
Agarose gel electrophoresis of the amplified V_L_, V_H_ and scFv gene of NP11-4 mAb. Lane M: molecular weight marker; Lane 1: V_L_ gene; Lane 2: V_H_ gene; Lane 3: scFv gene.

The V_H_ and V_L_ genes were amplified from the mRNA, which was isolated from the parental hybridoma cell line NP11-4. The PCR products were detected by 1% agarose gel electrophoresis (***Fig. 1***, Lanes 1 and 2). DNA sequencing demonstrated that the *V_L_* and *V_H_* genes were 396 bp and 366 bp in size, respectively. Using the primers V_H_F and V_L_R, the scFv gene was amplified by overlap PCR. The PCR product migrated as a 700bp fragment on an agarose gel (***Fig. 1***, Lane 3). DNA sequencing showed that a 762 bp band was obtained.

### Expression and purification of the scFv in *E. coli*

**Fig. 2:**
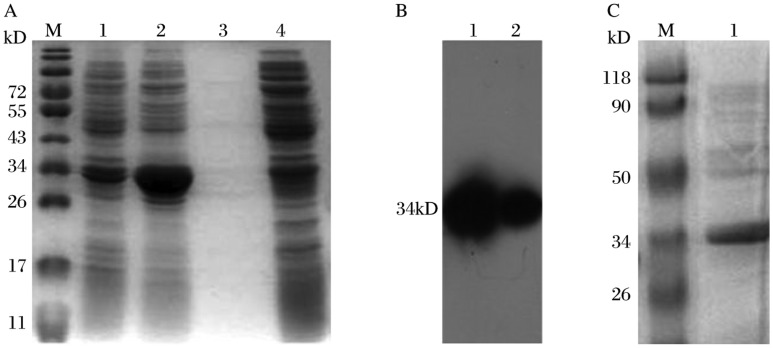
Expression and purification analysis. *E.coli* Top10F′ was transformed with the pBAD/gIII A-scFv vector. After induction, both soluble periplasmic proteins and insoluble proteins were extracted and analyzed by SDS-PAGE and Western blot. A: SDS-PAGE. Lane M: standard protein molecular weight marker; Lane 1: soluble periplasmic proteins; Lane 2: insoluble proteins; Lane 3: cultivation supernatant of the induced *E.coli* Top10F′; Lane 4: Negative control of *E.coli* Top10F′ without recombinant plasmid. B: Western blot analysis of scFv. Lane 1: insoluble proteins; Lane 2: soluble periplasmic proteins. C: SDS-PAGE analysis of purified scFv.

The scFv gene was cloned into the pBAD/gIII A vector, which encodes the 11-amino-acid product of the c-myc oncogene and 6×His. The recombinant plasmid pBAD/gIII A-scFv was transformed into *E.coli* Top10F′ and induced by the addition of 0.02% L-arabinose and 4% sucrose. Then the soluble periplasmic proteins and insoluble proteins were collected and analyzed by SDS-PAGE and Western-blotting assay. As shown in ***Fig. 2A***, a 34 kD protein was expressed, which was in agreement with the size of the scFv gene. Western blot analysis illustrated that scFv was expressed as both soluble and insoluble proteins (***Fig. 2B***). The soluble periplasmic proteins were collected and purified by a His-trap Ni-affinity column. The purified product was analyzed by SDS-PAGE; the result is demonstrated in ***Fig. 2C***. The yield of the purified scFv was approximately 0.8 mg per liter of culture medium.

### Construction of immunotoxin

The ultraviolet (UV) value detection was used to determine whether scFv and artesunate were conjugated. The UV absorbance of artesunate was about 210 nm, and the maximal absorption peak of scFv was 280 nm. After conjugation, the UV absorbance of the immunotoxin was about 238 nm, which was an average of the UV absorbance of scFv and artesunate. After washing by the final desalination column, the efficiency of conjugation was about 64.3%.

### Antigen-binding activity of scFv and immunotoxin

**Fig. 3:**
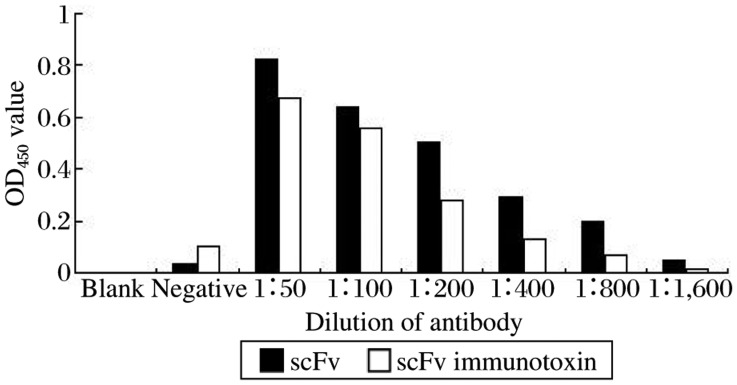
Comparison of antigen binding activity. The scFv and immunotoxin were used for quantitative analysis. Uninfected mouse serum was used as the negative control.

The binding activity of scFv and immunotoxin with SEA was detected by indirect ELISA. In each study, the same dose of antibodies was used to detect binding activity with SEA. With a reduced dose of antibodies, the value of *A*_450_ was decreased (***Fig. 3***). The results showed that chemical conjugation did not affect binding activity of the immunotoxin.

### Analysis of granuloma size

**Fig. 4:**
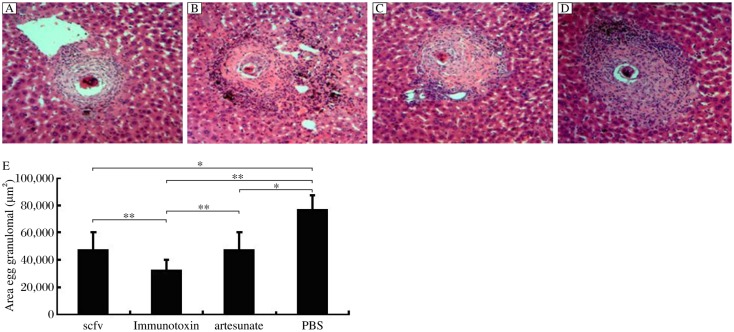
Area of egg granuloma analysis (×100). A: Immunotoxin group. B: Artesunate group. C: scFv group. D: PBS group. E: The immunotoxin group showed significant difference compared with other three groups(**P < 0.01). Compared with PBS group, the scFv group and the artesunate group also had significant difference (*P < 0.05).

All tissue sections were examined under a light microscope. One-Way ANOVA and SNK tests were performed to analyze the egg granuloma size. As shown in ***Fig. 4(A-D)***, the granulomas with a visible central egg were selected in each group. The size of granuloma was measured by computer-assisted morphometric analysis using the Image-Pro Plus software. The results showed that the size of granulomas in the PBS group was much larger than that of the other three groups. Moreover, compared with the scFv group and the artesunate group, the size of granulomas in the immunotoxin group showed a significant difference. However, there was no significant difference in the size of granulomas between the scFv group and the artesunate group (*P* > 0.05, ***Fig. 4E***).

### Hepatic fibrosis analysis

**Fig. 5:**
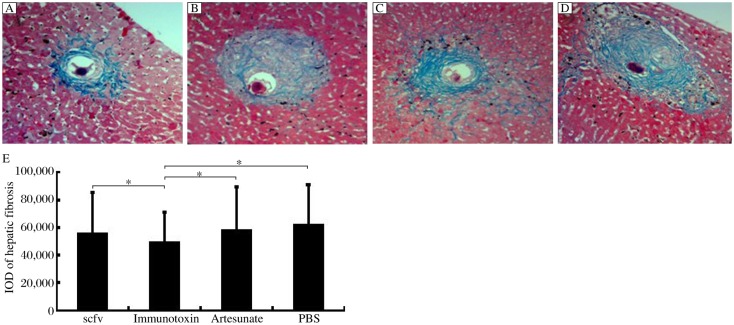
Hepatic fibrosis analysis. A-D: Blue staining was observed in the fibrotic lesions that were located around the *S. japonicum* eggs within the liver (×100). A: Immunotoxin group. B: Artesunate group. C: scFv group. D: PBS group. E: Immunotoxin could decrease the IOD of collagen levels obviously compared with other three groups (*P < 0.05).

The IOD of collagen levels in the liver tissue was analyzed by Masson staining (***Fig. 5 A-D***). One-Way ANOVA and SNK were also used to analyze the IOD of collagen levels. It was observed that fibrotic lesions were located around the *S. japonicum* eggs within the liver. Compared with the PBS group, the immunotoxin group could decrease the IOD of collagen levels obviously (*P* < 0.05). Moreover, compared with the scFv group and the artesunate group, there was a significant difference in the immunotoxin group. However, the scFv group and the artesunate group showed no significant difference when compared with the PBS group (*P* > 0.05, ***Fig. 5E***). The data indicated that the immunotoxin could significantly reduce hepatic fibrosis.

## DISCUSSION

Immunotoxin has been widely studied in cancer, such as gastric cancer[20], prostate cancer[21], malignant gliomas, and melanomas[22], but it has not been applied in the treatment of schistosomiasis so far. First-generation immunotoxins were composed of whole antibodies chemically conjugated to plant toxins (such as ricin or saporin) or bacterial toxins[23]. The limitation of the first-generation immunotoxins was their nonspecific toxicity and poor tissue penetration because of their larger molecular size[24].

ScFv was the smallest fragment with the original antigen-binding site[25]. It consists of the variable heavy (V_H_) and variable light (V_L_) chains joined by a flexible linker[26], and has displayed more advantages than whole antibodies or Fab fragments. Peptide linkers that join V_H_ and V_L_ chains together usually vary from 10-30 amino acids in length, and the most common linker is the (Gly_4_Ser)_3_[27]. Some studies have reported that variable regions could be linked in the V_L_-linker-V_H_ orientation or V_H_-linker-V_L_ orientation[28], and the latter is more commonly seen. The orientations can affect expression efficiency[29], stability, and antigen binding activity. In this study, an 18-hydrophilic amino acid flexible linker was designed in the V_L_ primer, and a scFv was generated in V_H_-linker-V_L_ form.

The scFv was expressed as soluble periplasmic proteins and insoluble proteins in temperatures ranging from 20°C-37°C. ScFv has been produced in *E. coli*, mainly as inclusion bodies. It should be washed with a denaturation buffer, followed by denaturation and refolding in a proper buffer to harvest the active proteins. However, the refolding process does not always produce completely native protein. It is advantageous to produce proteins soluble in *E. coli*. The scFv we developed was expressed as soluble proteins, and the appropriate temperature for soluble periplasmic protein expression was 23°C. After purification, the yield of soluble scFv was 0.8 mg/L.

In *S. japonicum* infections, a large number of eggs were incessantly produced from worms residing in the venules. The eggs elicited strong hypersensitivity reactions at the tissue sites where they were located, and the resultant destructive changes in the liver were well known as the primary etiology of schistosomiasis granuloma formation[30],[31]. The scFv that was generated from NP11-4mAb could bind to the membranes of cercariae, adult worms, and eggshells. It has few murine fractions and can reduce human antimouse antibody (HAMA) reactions, making it the best known candidate to construct immunotoxin.

Artesunate was considered as a vital cornerstone in the treatment and control of malaria in the past[32]. However, many laboratory experiments showed that artesunate was less toxic and highly effective against immature schistosomes. So it may be considered as an effective prophylactic antischistosomal drug[33],[34]. Hence, an immunotoxin containing anti-*S.japonicum* scFv and artesunate was constructed.

It is well known that drug resistance is unavoidable for any anti-infective compound. Recently, the existence of strains resistant to anti-*Schistosoma* agents was confirmed. The emergence of immunotoxin may solve the problem. It could target the destruction of immature and mature schistosomes and prevent their spawning, thus inhibiting granuloma formation and hepatic fibrosis. From the results of animal experiments, it appears that immunotoxin could significantly decrease granuloma size and hepatic fibrosis, compared with the other three groups. Immunotoxin may be used as a potential drug for treatment of schistosomiasis, but the specific mechanism of immunotoxin-inhibiting hepatic fibrosis requires further study.

In conclusion, scFv of NP11-4mAb against *S. japonicum* was obtained. After purification, the yield of soluble scFv was 0.8 mg/L. The immunotoxin, including scFv and artesunate, was constructed. Moreover, the immunotoxin was applied in the treatment of schistosomiasis japonica-induced liver fibrosis, and it had sufficient therapeutic effects. The statistical results demonstrated that immunotoxin could minimize the size of egg granuloma and reduce hepatic fibrosis significantly.
